# Definition of an Inflammatory Biomarker Signature in Plasma-Derived Extracellular Vesicles of Glioblastoma Patients

**DOI:** 10.3390/biomedicines10010125

**Published:** 2022-01-07

**Authors:** Chiara Cilibrasi, Thomas Simon, Marian Vintu, Christos Tolias, Mark Samuels, Nektarios K. Mazarakis, Murat Eravci, Nicolas Stewart, Giles Critchley, Georgios Giamas

**Affiliations:** 1 Department of Biochemistry and Biomedicine, School of Life Sciences, University of Sussex, Brighton BN1 9QG, UK; cc677@sussex.ac.uk (C.C.); simont@usc.edu (T.S.); marian.vintu@bsuh.nhs.uk (M.V.); ct440@sussex.ac.uk (C.T.); m.samuels@sussex.ac.uk (M.S.); m.eravci@sussex.ac.uk (M.E.); 2 Department of Translational Genomics, Keck School of Medicine, University of Southern California, Los Angeles, CA 90033, USA; 3 Department of Neurosurgery, University Hospitals Sussex, Brighton BN2 5BE, UK; giles.critchley@nhs.net; 4 Royal College of Surgeons in Ireland, D02 YN77 Dublin, Ireland; nm1@doctors.org.uk; 5 Department of Neurosurgery, Beaumont Hospital, D09 V2N0 Dublin, Ireland; 6 Centre for Stress and Age Related Diseases, School of Applied Sciences, University of Brighton, Brighton BN2 4GJ, UK; n.stewart@brighton.ac.uk

**Keywords:** glioblastoma, extracellular vesicles, liquid biopsy

## Abstract

Glioblastoma (GB) is an aggressive type of tumour for which therapeutic options and biomarkers are limited. GB diagnosis mostly relies on symptomatic presentation of the tumour and, in turn, brain imaging and invasive biopsy that can delay its diagnosis. Description of easily accessible and effective biomarkers present in biofluids would thus prove invaluable in GB diagnosis. Extracellular vesicles (EVs) derived from both GB and stromal cells are essential to intercellular crosstalk in the tumour bulk, and circulating EVs have been described as a potential reservoir of GB biomarkers. Therefore, EV-based liquid biopsies have been suggested as a promising tool for GB diagnosis and follow up. To identify GB specific proteins, sEVs were isolated from plasma samples of GB patients as well as healthy volunteers using differential ultracentrifugation, and their content was characterised through mass spectrometry. Our data indicate the presence of an inflammatory biomarker signature comprising members of the complement and regulators of inflammation and coagulation including VWF, FCGBP, C3, PROS1, and SERPINA1. Overall, this study is a step forward in the development of a non-invasive liquid biopsy approach for the identification of valuable biomarkers that could significantly improve GB diagnosis and, consequently, patients’ prognosis and quality of life.

## 1. Introduction

Glioblastoma multiforme (GB) is defined as a high-grade glioma and the most common intrinsic malignancy of the central nervous system (CNS). In the last decade, significant progress has been made in understanding the pathogenesis of gliomas. In 2016, the World Health Organization (WHO) updated the classification of gliomas and incorporated both histological and genetic/molecular parameters [1]. The fifth edition of the WHO classification of tumours of the CNS, published in 2021, further revised the GB definition as an *IDH* wild-type tumour presenting one or more of the following three genetic parameters: telomerase reverse transcriptase (*TERT)* promoter mutation, epidermal growth factor receptor (*EGFR*) gene amplification and combined gain of entire chromosome 7 and loss of entire chromosome 10 [2]. These molecular identities provide a more accurate prediction of response to treatment and survival rates [3]. In addition, further transcriptomic studies have allowed for a clear subtyping of GB tumours based on specific marker expression. GB is thus typically classified into three subtypes, namely, proneural, classical and mesenchymal, according to the different gene expressions of various biomarkers including platelet-derived growth factor receptor (*PDGFR*), neurofilament light (*NF1*), *EGFR* and *CD44* [4,5]. However, GBs are highly heterogenous, as these subtypes can co-exist in the same tumour bulk. Accordingly, recent single-cell RNA sequencing revealed that GB cells can exist in four main cellular states that recapitulate distinct neural cell types: neural progenitor-like (NPC-like) cells, oligodendrocyte progenitor-like (OPC-like) cells, astrocyte-like (AC-like) cells and mesenchymal-like (MES-like) cells [6].

Yet, despite significant progress in describing the molecular basis of the tumour pathogenesis and consecutive standardisation of classification and treatment, GB prognosis is still very poor [7,8]. GB usually recurs at or near the primary site within months of surgical removal [8]. GB patients have a median survival of 14–15 months with most patients dying within the first two years from the time of diagnosis [9,10].

The complex interactions between cancer cells and their surrounding microenvironment are amongst the parameters that make GB treatment challenging [11,12,13]. Extracellular vesicles (EVs) represent one of the means of such tight intercellular communication [14,15,16]. There has much interest in EVs for their potential roles in various normal and pathological conditions, while methods for their detection, concentration and analysis have substantially improved over the last 5 years [17,18]. Defined as lipid bilayer enclosed vesicles secreted by both normal and cancer cells, EVs are further identified as small EVs (sEVs) if smaller than 200 nm, while medium to large EVs (m/lEVs) are bigger than 200 nm [17]. Through transfer of proteins, nucleic acids and lipids, they are directly implicated in the constant crosstalk of GB cells with their tumour microenvironment (TME) [15,19,20,21]. GB-cell-derived EVs have, for instance, been observed to be involved in the transition of normal astrocytes into tumour-associated astrocytes, supporting tumour progression [22]. In return, EVs derived from stromal cells of the GB TME can influence tumour cells and impact on their aggressiveness [15,23].

Accordingly, we and others have observed that the cargo of sEVs can mirror the molecular background of their GB cell of origin, consequently providing crucial information regarding the associated molecular subtype, the tumour aggressiveness and the response to treatments [24,25,26,27,28,29,30].

For these reasons, EVs have gained increased interest over the last decade as potential biomarker candidates for diagnosis, disease recurrence and monitoring response to treatment in GB patients [31,32,33]. Currently, serial brain imaging is used for detecting tumour progression. However, this modality is challenging, as it is sometimes difficult to determine true progression from pseudo-progression [34]. On the other hand, circulating EVs have very short half-life in vivo and, therefore, can be used in identifying rapid changes in tumour progression [35]. This method could prove invaluable and perhaps identify tumour progression at a stage which is undetectable by conventional imaging.

In this study, we isolated sEVs from blood liquid biopsies of GB patients and characterised their proteomic cargo to identify potential candidate GB biomarkers. An inflammatory biomarker signature was described in sEVs from GB patients. Such application would be invaluable in future clinical practice for diagnosis and fine-tuning of GB treatment.

## 2. Materials and Methods

### 2.1. Patients and Clinical Samples

**Figure 1:**
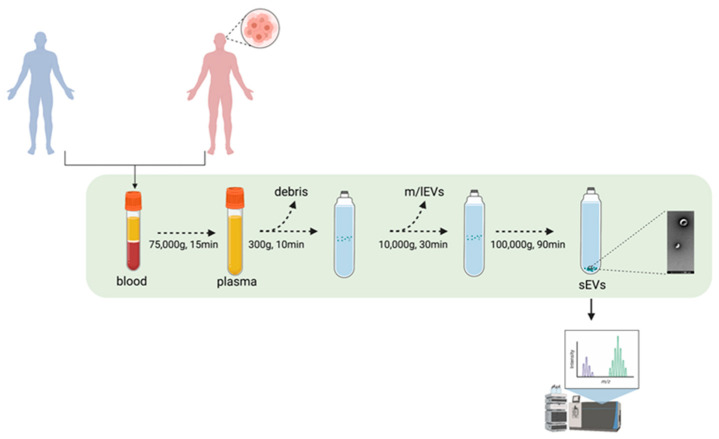
Experimental pipeline. sEVs from GB patients and healthy volunteers’ blood samples were isolated through UC. The proteomic content of the sEVs were deciphered using mass spectrometry. Figure was created using BioRender.com.

Healthy donors (*n* = 10) and consenting GB patients (*n* = 15) were enrolled at the Royal Sussex County Hospital (Brighton, UK) between July 2018 and February 2019. Ethical approval (REC reference: 18/EM/0071) was obtained for a prospective laboratory study. At the time of craniotomy, 5 mL of blood were taken from patients with histologically confirmed GB and available follow-up data. Healthy donors were blood donors matched for age and sex. The experimental design is reported in Figure 1.

### 2.2. Differential Ultracentrifugation for Concentration of Extracellular Vesicles

A total of 5 mL of blood was collected from each patient and healthy control. Plasma was isolated from the blood via ultracentrifugation (UC) at 75,000× *g* for 15 min. In turn, sEVs were concentrated by differential UC. Every step of the concentration protocol was performed at 4 °C. Plasma was pipetted into each UC tube and diluted to 20 mL in filtered sterile PBS. An initial 300× *g* centrifugation was performed for 10 min to discard any floating debris, followed by a 10 min centrifugation step at 2000× *g* to remove any further floating debris (Universal 320R centrifuge, Hettich, Tuttlingen, Germany). A 10,000× *g* UC step (Optima LE 80-k ultracentrifuge, Type 70 Ti rotor, polypropylene centrifuge 25 × 89 mm tubes, Beckman Coulter, Pasadena, CA, USA; full dynamic braking, k_adj_ = 15,638) was then performed for 30 min to remove potential large vesicles (m/lEVs). Finally, a first 100,000× *g* UC run was performed for 1 h and 30 min to pellet the putative sEVs (Optima LE 80-k ultracentrifuge, Type 70 Ti rotor, polypropylene centrifuge 14 × 89 mm tubes, Beckman Coulter, Pasadena, CA, USA; full dynamic braking, k_adj_ = 494). The UC pellet was then washed in filtered sterile PBS and centrifuged again for 1 h and 30 min at 100,000× *g* in order to discard contaminants. The final pellet was re-suspended in 100 µL filtered sterile PBS and immediately characterised through nanoparticle tracking analysis (NTA).

### 2.3. Nanoparticles Tracking Analysis (NTA)

Concentration and size of sEVs were determined using a NanoSight NS300 instrument and the NanoSight NTA 3.2 software (Malvern Panalytical, Malvern, UK). The following conditions were applied for the NTA analysis: temperature, 20–25 °C; viscosity, ~0.98 cP; camera type, sCMOS; laser type, Blue488; camera levels, either 14 or 15; syringe Pump Speed, 70 AU; 5 measurements of 60 s each were recorded. Graphs show the average of *n* = 10 healthy volunteers and *n* = 15 GB patients.

### 2.4. Coomassie Blue Staining

The sEV samples were loaded on 10% tris-glycine gels and run at 180 V and 40 mA for 100 min. The gels were then stained with Quick Coomassie Stain (Generon, Slough, UK) at room temperature overnight. Excess stain was removed through deionised water washes. Gels were viewed and captured by Criterion Stain Free Imager (Biorad, Hercules, CA, USA).

### 2.5. Western Blotting

Characterisation of the sEVs was performed through Western blotting by measuring the expression of EV membrane and cytosolic markers. Standard Western blotting protocol was performed as described before [36]. Briefly, protein concentration of the lysates was determined using the Pierce MicroBCA protein assay kit (Thermo Fisher Scientific, Waltham, MA, USA) and 10 μg of sEVs were loaded on the SDS gel. Proteins were then transferred onto a nitrocellulose blotting membrane (Thermo Fisher Scientific, Waltham, MA, USA) using the iBlot 2 dry blotting system (Thermo Fisher Scientific, Waltham, MA, USA). The membranes were blocked in TBS containing 0.1% (*v*/*v*) Tween 20 and 5% (*w*/*v*) BSA (Sigma–Aldrich, St. Louis, MO, USA) for 1 h before being incubated with the primary antibodies overnight at 4 °C. Primary antibodies used: anti-CD-9 (EXOAB-CD9A-1, 1:1000, System Biosciences, Palo Alto, CA, USA), anti-CD63 (EXOAB-CD63A-1, 1:1000, System Biosciences, Palo Alto, CA, USA), anti-CD81 (EXOAB-CD81A-1, 1:1000, System Biosciences, Palo Alto, CA, USA), anti-HSP70 (EXOAB-HSP70-1, 1:1000, System Biosciences, Palo Alto, CA, USA) and anti-GM130 (1:1000, Cell Signaling, Danvers, MA, USA). HRP-conjugated secondary anti-rabbit (1:5000, Cell Signaling, Danvers, MA, USA) or anti-mouse (1:5000, Cell Signaling, Danvers, MA, USA) antibodies were used. Chemiluminescent detection was performed using the SuperSignal West Pico PLUS Chemiluminescent Substrate (Thermo Fisher Scientific, Waltham, MA, USA). Emission was captured using the UVP ChemStudio Imaging Systems (Analityk Jena, Jena, Germany).

### 2.6. Transmission Electron Microscopy

Transmission electron microscopy (TEM) was performed on the putative sEV preparation in order to visualise and assess/confirm the size range of the vesicles as described before [37]. Samples were visualised using a JEOL JEM1400-Plus (Jeol, Tokio, Japan) (120 kV, LaB6) microscope equipped with a Gatan OneView 4K camera at 30× k magnification, and 10–15 pictures per grid were taken.

### 2.7. sEVs Analysis by ExoView

The EV markers’ expression was evaluated using the ExoView R100 platform (NanoView Biosciences, Brighton, MA, USA). 

Representative sEV samples from GB patients (*n* = 2) and healthy volunteers (*n* = 2) were analysed according to the manufacturer’s protocol. Briefly, diluted samples were incubated for 16 h at RT on ExoView Tetraspanin chips (EV-TETRA-C, NanoView Biosciences, Brighton, MA, USA), placed in a sealed 24-well plate. Chips contained spots printed with anti-CD63, anti-CD81, anti-CD9 or anti CD41a antibodies for EV populations characterisation. Mouse IgG1 matching isotype antibody was used as a control for non-specific sEV binding. Chips were then washed three times under gentle shaking and incubated for 1 h at RT with the ExoView Tetraspanin Labelling antibodies mix (EV-TC-AB-01, NanoView Biosciences, Brighton, MA, USA), containing 647 conjugated anti-CD63, 555 conjugated anti-CD81, and 488 conjugated anti-CD9 antibodies. The immunostained chips were washed three times in PBS, rinsed in filtered deionised water and dried. Chips were then imaged with the ExoView R100 reader (NanoView Biosciences, Brighton, MA, USA) using the ExoScan 2.5.5 acquisition software (NanoView Biosciences, Brighton, MA, USA).

### 2.8. Mass Spectrometry

In order to elucidate the protein content of the tumour-derived sEVs, mass spectrometry (MS) analysis was performed. To do so, a BCA assay was performed to determine the protein concentration of each sEV sample, and 100 ng were then loaded on an SDS-PAGE gel for protein separation. Following Coomassie blue staining, 5 slices/lane were then cut out of the gel and further processed for in-gel trypsin digestion and mass spectrometry run. De-staining was performed through 3 changes/washes with 50% acetonitrile (MeCN), 25 mM NH_4_HCO_3_ with 5 min of shaking between each change. Reduction and alkylation were performed, respectively, with 10 mM dithiothreitol (DTT) in 25 mM NH_4_HCO_3_ (45 min at 50 °C) and 50 mM chloracetamide and 25 mM NH_4_HCO_3_ (45 min in the dark at room temperature). Subsequently, 12.5 ng µL^−1^ trypsin (in 25 mM NH_4_HCO_3_) was added to the samples, followed by overnight incubation at 37 °C. The digest solution was then transferred to clean tubes. Next, 70% acetonitrile/5% trifluoroacetic acid was added to the gel pieces. Following 5 min of shaking, the supernatant was transferred to the corresponding clean tubes. A similar further extraction was repeated another two times to completely dehydrate the gel pieces and, consequently, to recover the rest of the peptides. The sample volume was reduced to 20 µL using a vacuum concentrator. Samples were then processed through a Q Exactive mass spectrometer (Thermo Fisher Scientific, Waltham, MA, USA) coupled to a Dionex Ultimate 3000 RSLCnano system (Thermo Fisher Scientific, Waltham, MA, USA). 

### 2.9. Proteomic Data Analysis

Protein identification and label-free quantification of proteins from MS and MS/MS raw data were performed using the MaxQuant software suit (version 1.6.12.0) (Max Planck Institute of Biochemistry, Planegg, Germany) with the implemented peptide search engine Andromeda [38] against a reference proteome database of Homo sapiens (Human/Uniprot proteome ID: UP000005640, Version 7 March 2021). Statistical evaluation of differentially expressed proteins between healthy volunteers (HV) and GB samples (EV) was performed with Welch’s *t*-test using the Perseus software suit (version 1.6.14.0) (Max Planck Institute of Biochemistry, Planegg, Germany). To facilitate statistical comparisons for proteins with little to no expression values in one of the compared groups, which was frequently the case in the HV group, missing values were substituted by an imputation of the lowest LFQ value from the entire data set.

Gene enrichment analysis of “biological process” and Vesiclepedia consultation were performed using the FunRich platform (http://www.funrich.org/) (last accessed 22 November 2021) [39].

### 2.10. The Cancer Genome Atlas and Clinical Proteomic Analysis Consortium Data

The mRNA expression levels derived from The Cancer Genome Atlas (Affymetrix HT HG U133A) were analysed using the Gliovis platform: http://gliovis.bioinfo.cnio.es (last accessed 22 November 2021) [40].

Protein expression analysis using data from the Clinical Proteomic Analysis Consortium (CPTAC) Confirmatory/Discovery data set was performed through the UALCAN web resource: http://ualcan.path.uab.edu/ (last accessed 30 December 2021) [41].

### 2.11. Statistical Analysis

Results are reported as the mean ± standard error of the mean (SEM). Unpaired *t*-tests were employed to determine the significance of the observed differences. Differences were considered statistically significant at *p* < 0.05 (95% confidence interval, * *p* < 0.05; ** *p* < 0.01; *** *p* < 0.001).

## 3. Results

### 3.1. Patients’ Clinical Characteristics

To verify the potential value of circulating sEVs as biomarkers for GB, plasma-derived sEVs from 15 GB patients and 10 healthy controls were isolated.

The GB patients were recruited between July 2018 and February 2019. The female-to-male ratio was 4:6, and the age at presentation was between 48 and 76 with a mean age of 59 (Table 1). Tumours were mainly located in either the temporal, frontal or occipital lobes. In some cases, the tumours affected more than two lobes (Table 1). Patient presentation included headache, seizure and focal neurological deficit. Detailed information about the clinical information on the GB patients enrolled in the study and the molecular features of the tumours can be found in Table 1 and Table S1, respectively.

### 3.2. Characterisation of sEVs Isolated from Peripheral Blood

Standardised and validated differential UC procedures were applied to concentrate sEVs from plasma derived from GB patients and healthy donors.

**Figure 2:**
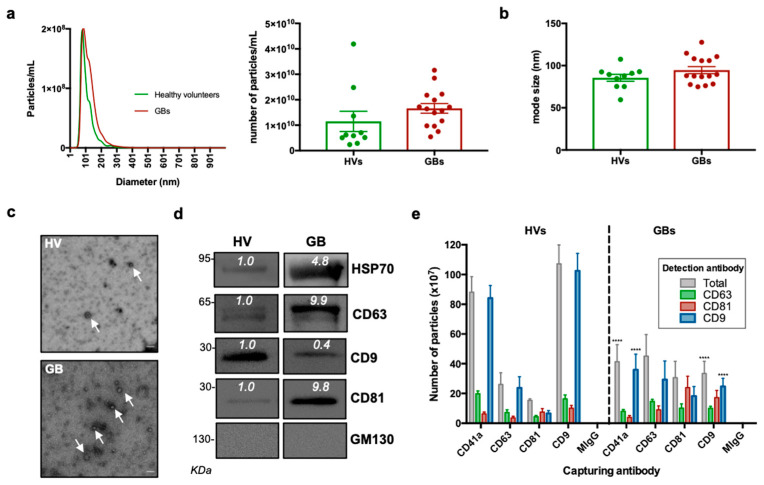
Characterisation of sEVs isolated from GB patients and healthy volunteers. (**a**) NTA of sEVs. The sEV suspensions from healthy volunteers (HV) (*n* = 10) and GB patients (*n* = 15) were diluted 1:50 and infused into a NanoSight NS300 instrument. Five captures of 60 s each were recorded. Particle concentration (particles/mL) and size (nm) were measured. The mean number of particles/mL ± SEM is shown. (**b**) Mode size (nm) ± SEM of sEVs is shown (healthy volunteers = 10; GB patients = 15). (**c**) Representative electron microscopy images of GB patient- and healthy volunteer-derived sEVs; 30k magnification was used. White arrows show sEVs. Scale bar = 100 nm. (**d**) Representative Western blots of GB patient- and healthy volunteer-derived sEVs, showing the presence of protein markers commonly associated with sEV subpopulations. The absence of the Golgi protein marker, GM130, indicates that non-EV cellular components were below the detection threshold. The same protein amounts of sEVs (10 μg) were loaded per lane. (**e**). ExoView analysis of sEV markers. sEV samples were incubated on microarray chips coated with the indicated antibodies. CD9-, CD63- and CD81-positive particles were detected after probing with a cocktail of fluorescent tetraspanin antibodies using the ExoView R100 platform. Results are the average of 2 healthy volunteer- and 2 GB patient-derived samples. For each sample, three technical replicates were performed. All data were adjusted for the dilution of the sample onto the chip. **** *p* < 0.0001.

The sEV size distribution and concentration were initially determined by nanoparticle tracking analysis. As shown in Figure 2a,b, the average total sEV concentration (particles /mL) and size modes were 1.15 × 10^10^ particles/mL and 86 nm for healthy volunteers and 1.66 × 10^10^ and 94 nm for GB patients. Of note, no statistically significant difference was observed in the sEV concentration and average size between GB patients and healthy controls.

Coupled to the NTA results, the electron microscopy documented the presence of lipid bilayer vesicles in the well-described size range of 50–150 nm (Figure 2c). The detection of sEV membrane-associated (i.e., CD9, CD63 and CD81) and cytosolic markers (HSP70) through Western blotting further confirmed the successful sEV concentration from the different samples, whereas the absence of the negative marker, GM130, indicated that non-EV cellular components were below the detection threshold (Figure 2d). The expression of sEV markers was also validated using the ExoView instrument (NanoView Biosciences, Brighton, MA, USA). The GB and healthy donor sEVs expressed all three tetraspanins and the platelet marker, CD41a, with varying co-expression of each (Figure 2e). The expression analysis performed with the ExoView instrument revealed a decrease in CD41a^+^ and CD9^+^ sEVs in the GB patient samples compared to the controls. On the other hand, as shown in the representative Western blots in Figure 2d, there was a slight increase in CD63^+^ and CD81^+^ sEVs in the GB patient samples compared to the controls.

### 3.3. Proteomic Analysis of GB sEV Cargo Provided a GB-Related Signature

**Figure 3:**
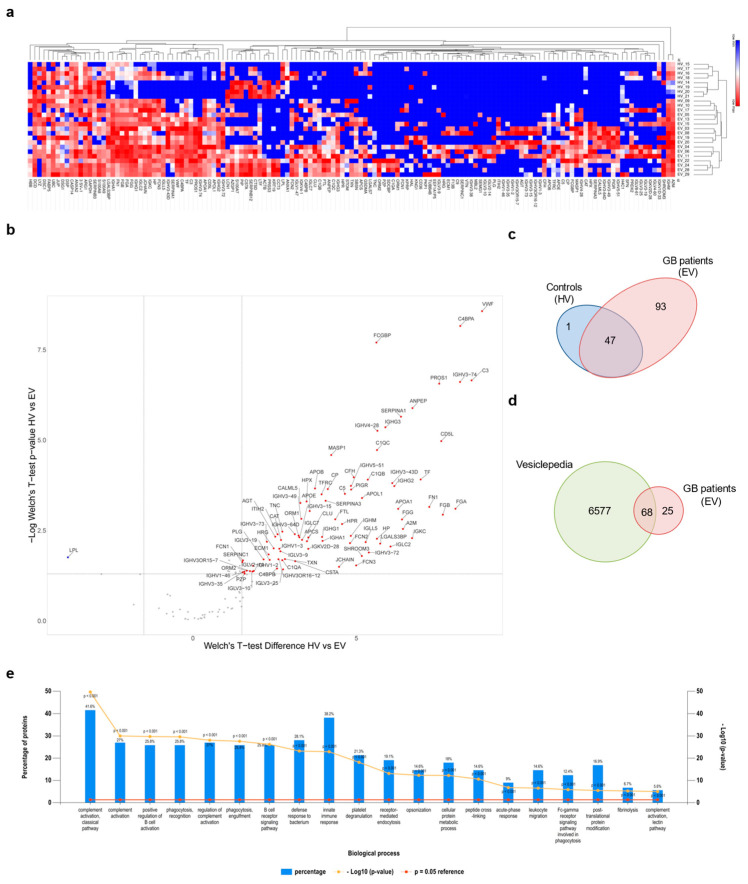
Proteomic analysis of sEV cargo. (**a**) Hierarchical clustering of protein expression in all experimental samples (i.e., healthy volunteers (HVs) and GB patients (EVs)) using Euclidian distance and average linkage, showing a heat map of protein expression for the respective samples (no protein expression in blue to high protein expression in red) and dendrograms for the hierarchical clustering of samples (horizontal dendrogram) and for proteins (vertical dendrogram), respectively. Cluster analysis was performed with the Morpheus online tool. (https://software.broadinstitute.org/morpheus, last accessed 22 November 2021) [42]. (**b**) Volcano plot showing the differentially expressed proteins between HVs and EVs with Welch’s *t*-test *p*-values < 0.05 and a log_2_ Welch’s *t*-test difference of at least 1.5 for upregulated proteins (red dots) and −1.5 for downregulated proteins (blue dots). A volcano plot was created with VolcaNoseR [43]. (**c**) Venn diagram showing the repartition of the identified MS hits between HVs and EVs. (**d**) Venn diagram of proteins enriched in GB-derived sEVs compared with proteins annotated in the Vesiclepedia database. (**e**) Gene enrichment analysis for “biological process” was performed based on MS hits using the FunRich platform. The 20 most significant processes identified are reported.

In order to obtain qualitative information about the protein cargo of GB circulating sEVs, a mass spectrometry analysis was performed on 10 healthy volunteer- and 15 GB patient-derived sEVs. A total of 141 proteins were identified with high confidence. Bioinformatic analysis was performed in order to identify statistically significant differences in protein expression between cancer and healthy donor plasma-derived sEVs. Hierarchical clustering of protein expression revealed that GB-derived sEVs presented a distinct signature (Figure 3a). Interestingly, 94 proteins were identified to be significantly differentially expressed between GB samples and healthy volunteers by performing a Welch’s *t*-test. One protein was specifically under-represented (i.e., lipoprotein lipase, LPL), while 93 proteins were specifically enriched in GBs (Figure 3b,c). Next, the set of GB-upregulated proteins were cross-referenced with a publicly available extracellular proteome database, showing that 68 hits were previously documented to exist in sEVs (Figure 3d). Further gene enrichment analysis revealed that biological processes linked with “complement activation”, “innate immune response”, “positive regulation of B-cell activation”, “phagocytosis recognition”, “platelet degranulation” and “B-cell receptor signalling pathway” were among the top ones identified in GB patient-derived sEVs compared to the healthy controls (Figure 3e).

**Figure 4:**
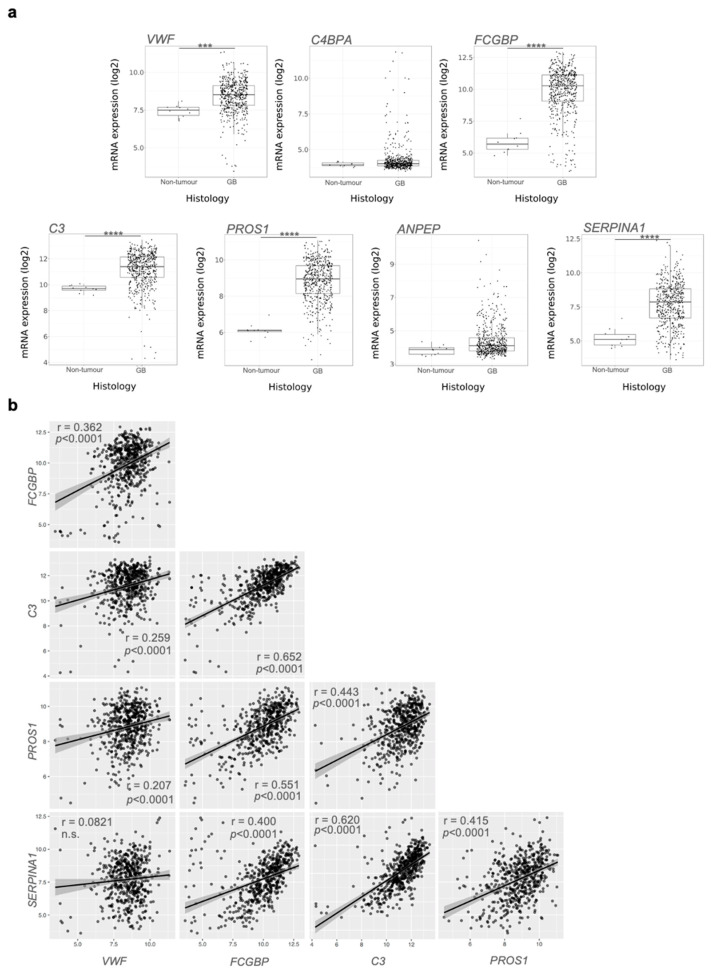
mRNA evaluation from The Cancer Genome Atlas data: (**a**) *VWF*, *FCGBP*, *C3*, *PROS1*, and *SERPINA1* showed significant mRNA upregulation in GB compared to healthy-derived samples. Pairwise *t*-test: *** *p* < 0.001, **** *p* < 0.0001. (**b**) Correlation of mRNA levels between the indicated genes. n.s.: statistically not significant.

mRNA expression data of 7 of the 10 most enriched protein were recovered from The Cancer genome Atlas (TCGA). The mRNA levels of *VWF*, *FCGBP*, *C3*, *PROS1*, and *SERPINA1* were upregulated (Figure 4a), and the expression of most of them was positively correlated in GB (Figure 4b). Similarly, pan-tumour protein expression evaluation using data recovered from the CPTAC data set [44] showed that those proteins were overexpressed in GB, although a correlation with other tumour types cannot be completely excluded (Figure S1). All these data suggest that sEVs cargo could mirror the landscape of the original tumour, characterised by abnormal vascularisation, coagulation and altered immune response, and that selective circulating sEV-derived proteins might be used as hallmarks for GB patients.

## 4. Discussion

Diagnosis of GB remains a clinical challenge. Current GB detection relies on symptomatic presentation of the tumour, magnetic resonance imaging and invasive tissue biopsy, which can delay the identification of the growing malignant mass [8]. Therefore, there is an urgent need in the development of diagnostic tools that would afford timely and non-invasive assessment of the disease in GB patients [15,45]. Plasma, serum, urine and cerebrospinal fluid (CSF) are biosources of tumour-associated EVs, containing biomarkers that reflect the biological landscape of GB, the state of the tumour, the disease progression and the response to cancer treatments [15,45,46]. In addition to their stability in biological fluids, EVs hold the capacity to protect and maintain the integrity of their content, preventing degradation and enabling its further study [16,46]. Therefore, we and others recently showed that the isolation of sEVs from plasma or any other biofluids and the analysis of their cargos are emerging as potential diagnostic and prognostic tools, allowing for the early detection and post-treatment surveillance of GB patients [24,33,47,48].

In the present study, we assessed circulating sEVs as potential carriers of GB biomarkers, through comparison of the proteomic content of sEVs derived from blood biopsies of GB patients and healthy donors.

Here, we report the successful concentration of sEVs from plasma via differential UC, as validated through NTA, TEM and detection of known sEVs markers such as CD9, CD63, CD81 and HSP70. In addition, further analysis of the proteomic content revealed that most of the detected proteins have been previously reported in sEVs [49]. This confirmed the reliability of standard sEV concentration methods for diagnostic purpose when working with biofluids [17].

Concentration and size of sEVs derived from GB patients’ biofluids were similar to those of healthy donors. Other groups contrastingly reported higher plasma-derived sEV concentration in GB patients compared to healthy donors or other CNS tumour patients [33]. However, in a recent extended analysis of serum sEVs from patients with distinct CNS tumours performed on 96 samples, Dobra et al. observed no statistical differences in concentration or size. Such discrepancies among studies could be due to the variations in cohort numbers or methods. Yet, as mentioned by Dobra et al., other non-neoplastic disorders can also lead to increased concentrations of circulating sEVs, supporting our findings that concentration of plasma-derived sEVs is not a good indicator of malignant disease, let alone a GB-specific marker [50].

The protein cargo of plasma sEVs in GB patients and healthy controls have been characterised through an MS-based proteomic analysis to identify differentially expressed sEV-associated proteins as potential GB biomarkers.

Our proteomic analysis revealed that the content of plasma sEVs derived from GB patients was distinct from healthy volunteers-derived sEVs and showed enrichment for biological processes mainly associated with complement activation, immune response and B-cell activity, suggesting that sEVs collected from GB patient plasma show a specific “inflammatory” molecular profile [33]. Our data corroborate reports by other groups that identified a similar molecular signature in GB circulating sEVs [33,51]. Accordingly, among the most abundant protein candidates exclusively expressed in GB patient-derived sEVs, VWF, C3, FCGBP, PROS1 and SERPINA1 were further validated as potential GB-specific circulating markers through analysis of the available TCGA data.

In agreement with the present data, preoperative VWF plasma levels have been indicated as a potential prognostic marker in GB patients, with high expression linked to poor survival [52]. Further supporting our findings, VWF was included in the “GB EV protein signature” defined by Osti et al. in their cohort of GB patients’ plasma samples [33]. In addition, high sEV-VWF levels could also be linked to the hypervascularised nature of GB [13,20,53]. Previous studies accordingly reported a direct relationship between VWF levels in microvessels and different grades of astrocytomas [54]. VWF has also been identified as a potential circulating marker for tumour angiogenesis in other types of cancer [55].

As for VWF, components of the complement cascade, such as C3, have also been observed by others in sEVs derived from GB patients’ liquid biopsies [33,51]. As reported by Greco et al., the complement cascade was also one of the most enriched signalling in serum sEVs in a murine GB model [56].

Despite the ambivalent function of the complement system in different types of cancers [57], high expression of complement-related genes has been associated with poor prognosis in GB [58]. Moreover, C3 upregulation has been reported to be involved in recruiting myeloid-derived suppressor cells, maintaining the glioma stem cells pool and sustaining the neo-angiogenesis process [59].

Initially reported as an important component of immunological mucosal defences [60], FCGBP gained more interest recently due to the fact of its variable expression and contradictory roles in different types of malignancies, where it seems to be involved in the immune response associated with cancer development [61,62,63,64]. Of note, it has been shown to be a metastasis-associated gene in *IDH* wild-type gliomas [65] and a tumour antigen in low-grade gliomas [61].

Another immune mediator enriched here in the sEVs derived from GB patients was PROS1, a ligand of tumour-associated macrophage (TAM) receptors, which has immunosuppressive and carcinogenic roles in GB that have widely been accepted [66]. Interestingly, Sadahiro et al. demonstrated that PROS1 was secreted by TAMs/microglia in the perivascular area of mesenchymal GB tissues and bound to AXL on GB cells to contribute to the growth of aggressive tumours [67].

Finally, SERPINA1 is a key anti-inflammatory player reported to promote invasiveness and progression in a wide range of cancers [68,69,70,71,72] and to be a prognostic marker of poor overall survival in high-grade gliomas [73]. Interestingly both FCGBP and SERPINA1 have been proposed as GB-associated genes due to their upregulation in primary and secondary GBs compared to lower-grade astrocytomas [74].

Variations in the major players of the innate and adaptive immune system have been found in cancers, including GB, which ultimately leads to immune evasion, supporting cancer expansion and recurrence [75]. Therefore, the “inflammatory” biomarker signature we identified in the plasma sEVs of GB patients could be due to the immune and vascular perturbations taking place in the TME in reaction to cancer development [12,13,15,76].

Our present data highlight the need for moving away from single biomarker identification to describing specific protein expression patterns or signatures for more accurate diagnosis [50]. Along those lines, and as we reported in cell lines in a previous study, our results also revealed that expression of constitutive sEV markers, including tetraspanins CD9, CD63 and CD81, could differ between patient and control samples and, consequently, provide further indication about a specific tumour subtype or progression [24,77,78]. Accordingly, we observed that CD63 and CD81 expression increased in sEVs from GB patients compared to controls. As they have repeatedly been associated with tumour progression and immune regulation, both tetraspanins could also be good indicators of the inflammatory response that takes place in reaction to GB development [24,79,80,81].

Overall, our present report represents a further proof of concept for the use of sEV-associated biomarkers in liquid biopsies for GB detection [33,50,51]. Strikingly, despite the use of peripheral blood, all potential markers exclusively identified in patient samples here had already been specifically linked with either GB diagnosis, GB prognosis or GB-associated signalling, including communication with the surrounding TME. Yet, we will first need to validate the trends we observed in larger cohorts of plasma-derived sEVs, with the aim to further establish the diagnostic and clinical value associated with the “inflammatory” molecular signature described here. In the same way, measuring the expression of the present signature in the original GBM tumour tissues would also be valuable. Doing so would also allow to test if the content of sEVs strictly mirrors the proteomic profile of the original tissue or if the present signature is detectable exclusively in plasma-derived sEVs. Moreover, in future studies, proteomic analysis should be assessed during patients follow up to correlate the observed profile with MRI data, treatment, recurrence, molecular and clinical features [20,82]. The content of plasma-derived EVs from patients with other disorders associated with inflammatory processes or from patients with other tumour types, such as low-grade glioma, will be investigated and compared to our GB data. Such analysis would allow to further assess the specificity of the present signature for GB diagnosis. In the same way, it would be useful to couple proteomic investigation with transcriptomic and metabolomic profiles, to further validate sEVs as carrier of specific and reliable GB biomarkers and unveil a comprehensive -omic GB signature. Ultimately, future studies should also aim to decipher distinct sEV-omic signatures specific to each known GB subtype, namely, classical, proneural and the most aggressive mesenchymal subtype.

Altogether, our study is a step forward in the development of an accurate, non-invasive and time-saving tool for GB liquid biopsies that would significantly improve diagnosis and accurately define prognosis as well as provide best patient care.

## Figures and Tables

**Table 1:** GB patients’ clinical characteristics.

Study Number	Age	Gender	Lobe Affected	Surgery	Oncological Treatment	Recurrence	Months to Death
EV003	57	F	Left parietal–occipital–temporal	40–50% debulking	high-dose RT	yes	7
EV004	60	F	Right occipital	90% debulking	radical chemoRT	no	7
EV005	72	M	Right occipital	95% debulking	radical chemoRT; adjuvant TMZ: CCNU	yes	19
EV006	76	M	Left frontal	100% resection	n/a	no	1
EV008	48	F	Left temporal	100% resection	high-dose palliative RT	yes	8
EV011	64	M	Right frontal	100% resection	radical chemoRT; adjuvant TMZ	no	alive
EV013	65	M	Right parietal	70% resection	radical chemoRT; adjuvant TMZ	yes	12
EV015	50	F	Right temporal	90% resection	radical chemoRT; adjuvant TMZ	yes	11
EV017	64	M	Left temporal–occipital	70% resection	high-dose palliative chemoRT	yes	6
EV019	71	M	Left temporal	80–90% resection	high-dose RT	yes	2
EV020	55	M	Right temporal	80% resection	n/a	no	2
EV022	66	F	Right frontal	90–95% resection	radical chemoRT	yes	11
EV024	60	M	Right temporal–parietal	80% resection	high-dose RT	yes	10
EV028	53	F	Right frontal	90–95% resection	high-dose palliative RT	no	4
EV029	71	M	Left temporal	100% resection	radical chemoRT	no	alive

M, male; F, female; chemoRT, chemo-radiotherapy; RT, radiotherapy; TMZ, temozolomide; CCNU, lomustine; n/a, not available.

## Data Availability

All relevant data are available from the authors upon request. The mass spectrometry proteomics data were deposited at the ProteomeXchange Consortium via the PRIDE partner repository with the data set identifier.

## Supplementary Materials

The following are available online at https://www.mdpi.com/article/10.3390/biomedicines10010125/s1, Table S1: Tumour molecular features; Figure S1: Pan-tumour protein expression levels from the Clinical Proteomic Tumor Analysis Consortium (CPTAC) Confirmatory/Discovery data set.
