# Supplementary material for: Definition of an Inflammatory Biomarker Signature in Plasma-Derived Extracellular Vesicles of Glioblastoma Patients
Source: Biomedicines. 2022 Jan 7;10(1):125. doi: 10.3390/biomedicines10010125 (PMC8773644; doi:10.3390/biomedicines10010125)
Supplement: Supplementary file 1 [file biomedicines-10-00125-s001.zip › biomedicines-1490796-supplementary.pdf]

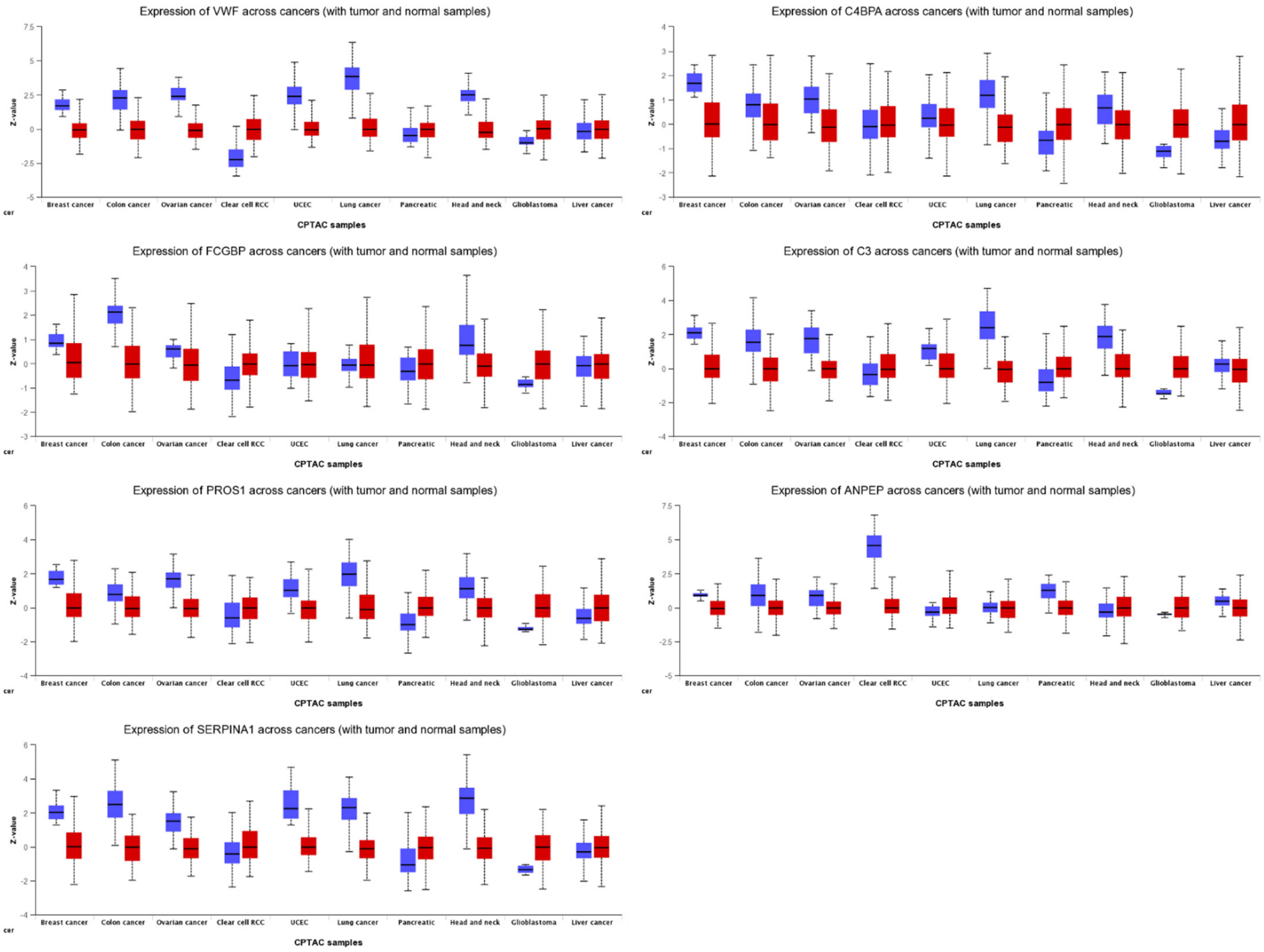

Figure S1. Pan-tumour protein expression levels from the Clinical Proteomic Tumor Analysis Consortium (CPTAC) Confirmatory/Discovery dataset. Protein levels have been recovered using the UALCAN web resource. 7 of the 10 most enriched protein in sEV samples from GB patients are overexpressed in GB.

Table S1. Tumour molecular features.

Study Number	1p19q Codeletion (LOH)	*MGMT* Methylation	*IDH1/ IDH2* Mutation	Chr 7p (*EGFR*)	Chr 10q (*PTEN*)	*ATRX*	*TERT*	Histone *H3F3A*	*BRAF*
EV003	Retained	Methylated 0-5%	No mutation	Weak amplification	No LOH	Retained	Mutation	No mutation	No mutation
EV004	Retained	Methylated 10-25%	No mutation	No amplification	No LOH	Retained	Mutation	No mutation	No mutation
EV005	Retained	Methylated 0-5%	No mutation	No amplification	LOH	Retained	Failed	No mutation	No mutation
EV006	Retained	Unmethylated 0%	No mutation	No amplification	LOH	Retained	Failed	No mutation	Mutation
EV008	Retained	Methylated 10-25%	No mutation	No amplification	No LOH	Loss of expression	Failed	No mutation	No mutation
EV011	Retained	Unmethylated 0%	No mutation	No amplification	No LOH	Retained	Mutation	No mutation	No mutation
EV013	Codeleted	Unmethylated 0%	No mutation	No amplification	LOH	Retained	Failed	No mutation	No mutation
EV015	Retained	Unmethylated 0%	No mutation	No amplification	LOH	Retained	Failed	No mutation	No mutation

EV017	Retained	Methylated >25%	No mutation	No amplification	LOH	Retained	Mutation	No mutation	No mutation
EV019	Retained	Methylated >0-5%	No mutation	No amplification	LOH	Retained	Mutation	No mutation	Failed
EV020	Retained	Methylated >25%	No mutation	No amplification	no LOH	Failed	Mutation	No mutation	No mutation
EV022	Retained	Unmethylated 0%	No mutation	No amplification	LOH	Retained	Mutation	No mutation	No mutation
EV024	Retained	Unmethylated 0%	No mutation	No amplification	no LOH	Retained	Mutation	No mutation	No mutation
EV028	n.a.	Unmethylated 0%	No mutation	No amplification	n.a.	Retained	Mutation	No mutation	No mutation
EV029	n.a.	Unmethylated 0%	No mutation	No amplification	n.a.	Retained	No mutation	No mutation	No mutation
